# Impact of Retinoic Acid on Immune Cells and Inflammatory Diseases

**DOI:** 10.1155/2018/3067126

**Published:** 2018-08-09

**Authors:** Luana de Mendonça Oliveira, Franciane Mouradian Emidio Teixeira, Maria Notomi Sato

**Affiliations:** Laboratory of Dermatology and Immunodeficiencies, LIM-56, Department of Dermatology, School of Medicine, University of São Paulo, Institute of Tropical Medicine of São Paulo, São Paulo, SP, Brazil

## Abstract

Vitamin A metabolite retinoic acid (RA) plays important roles in cell growth, differentiation, organogenesis, and reproduction and a key role in mucosal immune responses. RA promotes dendritic cells to express CD103 and to produce RA, enhances the differentiation of Foxp3^+^ inducible regulatory T cells, and induces gut-homing specificity in T cells. Although vitamin A is crucial for maintaining homeostasis at the intestinal barrier and equilibrating immunity and tolerance, including gut dysbiosis, retinoids perform a wide variety of functions in many settings, such as the central nervous system, skin aging, allergic airway diseases, cancer prevention and therapy, and metabolic diseases. The mechanism of RA is interesting to explore as both a mucosal adjuvant and a combination therapy with other effective agents. Here, we review the effect of RA on innate and adaptive immunity with a special emphasis on inflammatory status.

## 1. Introduction

Vitamins are essential components of diet and are essential for the maintenance of various biological processes. For example, vitamin A, through its active metabolite, retinoic acid (RA), acts in several biological conditions, such as embryonic development, hormone function, the maintenance and modulation of the immune response, and the homeostasis of epithelial tissues and mucosa [1, 2].

Vitamin A is obtained through diet, and its deficiency, especially in childhood, increases the morbidity and mortality risk from infectious diseases, especially diseases of the gastrointestinal and pulmonary tracts, causes blindness and anemia, and impairs vaccine responses [1, 3]. In low-income countries, children receive insufficient amounts of vitamin A during breastfeeding and childhood, making vitamin A deficiency a public health problem. Studies have shown that vitamin A supplementation reduces the mortality rate by 24% among children aged 6 months to 5 years [4]. For this reason, the World Health Organization (WHO) recommends vitamin A supplementation for infants and children aged 6–59 months in underdeveloped countries [5].

Indeed, after the absorption and metabolization of vitamin A into RA in the gut, RA plays critical roles in the mucosal immune response as a regulatory signal in the intestinal mucosa by promoting Foxp3 regulatory T cell differentiation [6] and immunoglobulin (Ig) A production [7]. In addition, RA induces the homing of innate immune cells, such as innate lymphoid cells (ILCs) [8] besides regulatory and effector T and B cells, to the gut [9–11]. During infections, RA can induce the production of proinflammatory cytokines by dendritic cells (DCs), promoting the differentiation of effector T cells and the protection of the mucosa [12]. Thus, RA is crucial for maintaining homeostasis at the intestinal barrier and equilibrating immunity and tolerance. Due to the extensive role of RA in immune cells and the immune response, reducing mortality in children by vitamin A supplementation may be possible [4].

In addition, due to its regulatory activity, RA has been shown to play an important role in the control of inflammatory diseases not only in the intestine [13, 14] but also in other tissues, such as the central nervous system [15–17] and pulmonary mucosa [18, 19].

Therefore, the roles of RA in the immune system, that is, both maintaining mucosal and epithelial homeostasis and contributing to anti-inflammatory function, are addressed in this review. The focus is on the role of RA in inflammatory responses, such as responses to inflammatory skin, intestinal, and airway diseases and its impact on immune cells. However, first, we discuss the metabolization of vitamin A into RA and its signaling pathways.

## 2. RA Metabolism and Signaling

**Figure 1:**
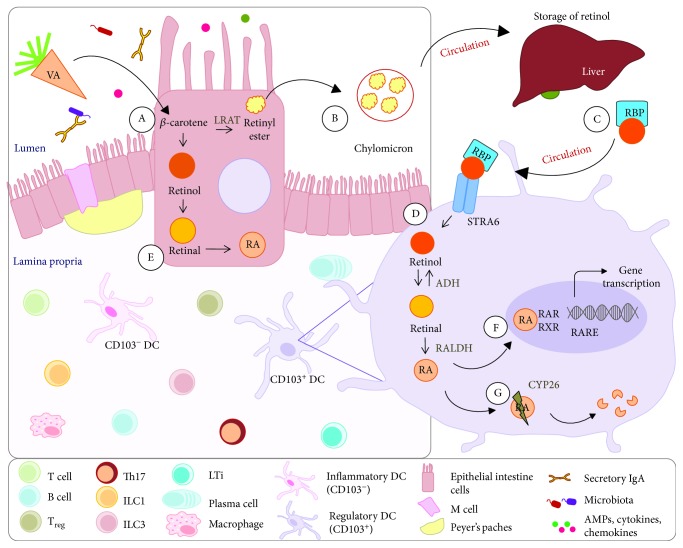
RA metabolism and signaling. (A) Vitamin A and its precursors (*β*-carotene) obtained from diet are absorbed by intestinal epithelium cells and esterified in retinyl esters by the enzyme lecithin retinol acyltransferase (LRAT). (B) Retinyl esters are packed with chylomicrons and enter general circulation where they are captured by hepatocytes and stored as retinol. (C) The retinol binds to retinol binding protein (RBP) in the liver and is carried through the bloodstream. This complex is recognized via the stimulated by retinoic acid 6 (STRA6) receptor, which mediates the absorption of extracellular retinol to cytosol. (D) After uptake, RA is generated from retinol by two sequential reactions. First, retinol is oxidized into retinal by enzyme alcohol dehydrogenase (ADH). Subsequently, in CD103^+^ DCs, retinal is oxidized by the enzyme retinal dehydrogenase (RALDH) to generate RA. (E) Intestinal epithelium cells can also metabolize vitamin A after absorption into retinal and RA, which can be directly released into the intestinal mucosa. (F) RA interacts with nuclear receptors, such as the retinoic acid receptor (RAR) and retinoid receptor X (RXR), to regulate the transcription of several target genes by binding the retinoic acid-responsive elements (RAREs) in DNA. (G) Control of the RA concentration in tissues is performed by a group of enzymes that belong to the cytochrome P450 family 26 (CYP26), which catalyzes RA present in the cytosol to generate the oxidized forms.

Vitamin A is obtained from diet though the consumption of foods containing vitamin A precursors (mainly *β*-carotene) and vitamin A in the form of retinyl esters, which are derived from plant and animal food, respectively [20]. Vitamin A and its precursors are absorbed in the intestine by intestinal epithelium cells, and the vitamin A precursors are esterified in retinyl esters by the enzyme lecithin retinol acyltransferase (LRAT). Retinyl esters are packed with chylomicrons and enter general circulation [21] (Figure 1). In the systemic circulation, the chylomicrons undergo the action of the lipoprotein lipase enzyme, resulting in their capture by hepatocytes and hydrolysis to retinol. Retinol is stored in the liver, mostly in hepatic stellate cells (HSCs) [1].

When RA is needed by the organism, the formed retinol binds retinol-binding protein (RBP) in the liver and is carried through the bloodstream [22]. This complex is recognized via the stimulated by retinoic acid 6 (STRA6) receptor, which mediates the absorption of extracellular retinol to cytosol [23]. However, the STRA6 receptor is only essential for maintaining RA homeostasis in the eye; therefore, other mechanisms are likely involved in the uptake of retinol into other tissues [24, 25].

After uptake, RA is generated from retinol by two sequential reactions. In the first reversible reaction, retinol is oxidized into retinal by the ubiquitously expressed enzyme alcohol dehydrogenase (ADH) [20]. Subsequently, in intestinal epithelium cells, DCs and macrophages associated with mesenteric lymph nodes (mLNs) and Peyer's patches (PPs), retinal is oxidized by the enzyme retinal dehydrogenase (RALDH) to generate RA [1]. There are three isoforms of RALDH (RALDH1, RALDH2, and RALDH3) [21], and their expression is tightly regulated and limited in the cells mentioned above. Thus, RALDH is considered the main enzyme that defines the populations of cells that are capable of producing RA [20]. Intestinal epithelium cells can also metabolize vitamin A after absorption into retinal and RA, which can be directly released into the intestinal mucosa [21].

RA can be generated in multiple forms as *all-trans*, *9-cis*, and *13-cis* RA [26, 27]; however, *all-trans* RA (atRA) is physiologically the most abundant [28]. RA interacts with nuclear receptors, such as the retinoic acid receptor (RAR) and retinoid receptor X (RXR), to regulate the transcription of several target genes [10, 29] by binding the retinoic acid-responsive elements (RAREs) in DNA [30]. These receptors form heterodimers; RAR comprises three major isoforms (*α*, *β*, and *γ*) that interact with all forms of RA, whereas RXR, which also has the *α*, *β*, and *γ* isoforms, mainly interacts with *9-cis* RA [31]. RA can also signal through peroxisome proliferator-activating receptor beta (PPAR-*β*) when it forms a heterodimer with RXR, which may be important for lipid metabolism and glucose homeostasis [1]. In addition, chicken ovalbumin upstream promoter-transcription factor II (COUP-TFII) [32] and hepatocyte nuclear factor 4 (HNF-4) [33] receptors can forms a heterodimer with RXR and become low-affinity retinoic acid receptors. Similar to PPAR-*β*, their signals are important for lipid metabolism and glucose homeostasis [32].

Control of the RA concentration in tissues is performed by a group of enzymes that belong to the cytochrome P450 family 26 (CYP26), including subfamilies A1, B1, and C1 (CYP26A1, CYP26B1, and CYP26C1), which catalyze RA present in the cytosol to generate the oxidized forms (*5*,*8-epoxy* RA, *4-oxo* RA, *4-hydroxy* RA, and *18-hydroxy* RA) [34, 35]. The action of these enzymes prevents RA accumulation in the organism and maintains optimal physiological RA concentrations for the best performance.

## 3. Effects of RA on Immune Cells

**Figure 2:**
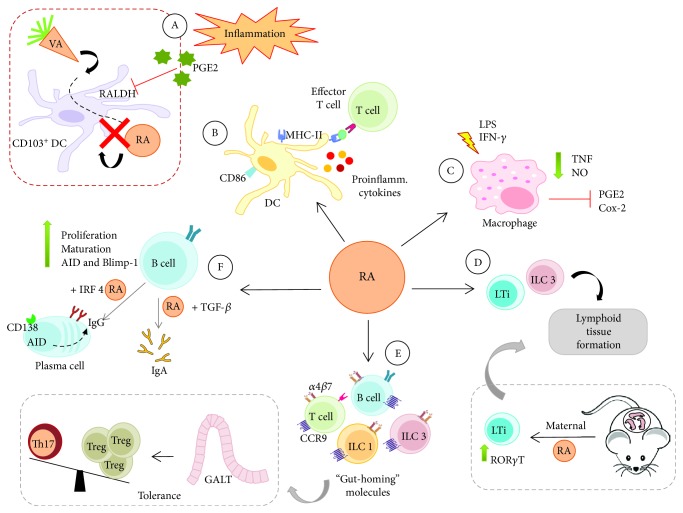
Role of RA in immune cells. RA can act on different cells of both the innate and adaptive immune systems exerting local action at mucosal sites and systemic action, which simultaneously, depending on where the RA-producing cells, mainly CD103^+^ DCs, are located when it releases the RA. (A) However, in an inflammatory environment (red box), PGE2 released during the inflammatory response inhibits the RALDH enzyme that is required for RA synthesis. When RA is released, it acts as follows: (B) RA together with proinflammatory cytokines contributes to the activation of DCs and the generation of effector T cells; (C) RA promotes macrophage modulation, inhibiting inflammatory mediators and the release of TNF and NO; (D) RA also activates ILC3, especially LTi cells, which are required for the formation of lymphoid tissue, including during fetal development; (E) RA induces expression of the molecules *α*4*β*7 and CCR9 in lymphocytes and ILCs and the homing of these cells into the intestine and promotes the balance of Th17/Treg cells in the GALT, assuring tolerance, but is also able to induce Th17 in the presence of infection and inflammation; and (F) RA promotes the activation of B cells and their differentiation into Ab-secreting plasma cells.

RA can act on different cells of both the innate and adaptive immune systems (Figure 2), exerting local action at mucosal sites, mainly in the intestinal mucosa, and systemic action. In addition, RA plays a key role in the maintenance of immune homeostasis during inflammatory responses.

### 3.1. Tolerogenic Effect of RA on DCs and Macrophages

The balance between tolerance and effector responses is mainly regulated by antigen-presenting cells (APCs), especially DCs [36]. DCs in peripheral organs are characterized by the expression of CD103 and CD11b molecules [37]. RA can regulate the differentiation of bone marrow DC precursors (pre-DCs) into premucosal DC (pre-*μ*DCs) by expression of gut-trafficking receptor *α*4*β*7 and gives rise to intestinal CD103^+^CD11b^+^ DC, in mice [38].

Tolerogenic CD103^+^ DCs, which are located mainly in the lamina propria of the small intestine and gut-associated lymphoid tissue (GALT), such as PPs and mLNs [39, 40], are responsible for the maintenance of homeostasis. This type of DC can promote the generation of Foxp3^+^ regulatory T cells and the migration of regulatory and effector cells to the GALT [9–11]. The migration of T and B cells is mediated by CD103^+^ DCs due to their ability to synthesize RA [10, 41] as these cells have a high expression of the RALDH1 and RALDH2 enzymes, which are responsible for the conversion of retinal to RA; thus, these cells are the main synthesizers of RA [42].

Other RALDH^+^ DC populations that also produce RA are mainly located at mucosal interfaces, such as the skin, the lungs, and the corresponding draining lymph nodes [43, 44]. At the moment of antigen presentation in secondary lymphoid organs, RALDH^+^ DCs (mainly CD103^+^ DCs) release RA, which can freely diffuse across the cell membrane of the target cell. Then, RA signaling via the RAR*α* receptor regulates the transcription of the promoter regions of the *α*4 gene subunit of *α*4*β*7 integrin and the CC chemokine receptor 9 (CCR9) gene on target cells [10], promoting the synthesis and expression of gut-trafficking receptors *α*4*β*7 and CCR9 in the cellular membrane. *α*4*β*7 and CCR9 can interact with mucosal vascular addressin cell adhesion molecule 1 (MAdCAM-1) and CC chemokine ligand 25 (CCL25), respectively [41, 45]. MAdCAM-1 is present in the venules of mLNs and PPs, while CCL25 is produced by intestinal epithelial cells; thus, RA imprints gut-homing specificity on immune cells [8, 40, 41, 45, 46].

However, not all DCs express the RALDH enzyme, such as inflammatory DCs, which can infiltrate or develop in the gut during inflammation due to chemokines and cytokines secreted by resident cells during the inflammatory process [47, 48]. In contrast to CD103^+^ DCs, the proinflammatory CD103^−^ DC population promotes the differentiation of interferon-gamma- (IFN-*γ-*) producing T cells and produces proinflammatory cytokines, such as tumor necrosis factor-alpha (TNF-*α*) and interleukin- (IL-) 6, suggesting that these subsets of DCs can play a distinct role in promoting effector T cell response in the gut [36, 49].

Some inflammatory factors may influence RALDH expression, such as prostaglandin E2 (PGE2), which is produced by peripheral stromal cells and suppresses the differentiation of RA^+^ DCs by directly antagonizing RALDH expression [50]. In addition, DCs that infiltrate the gut during inflammation do not acquire RALDH activity, which is required for RA synthesis. These inflammatory DCs express E-cadherin and the CD103 receptor, accumulate in the mesenteric lymph nodes and the inflamed colon, exhibit high expression of Toll-like receptors (TLRs) and produce cytokines IL-6 and IL-23, enhancing inflammation [47].

Mice with a vitamin A-deficient diet (VAD) exhibit reduced expression and activity of the RALDH enzyme in intestinal DCs, which is essential for the regulation of immune and inflammatory responses [51]. Others factors, including GM-CSF, IL-4, IL-13, and TLR ligands 2 and 5, may induce the *in vitro* expression of RALDH [52, 53], suggesting that the local microenvironment is able to modulate RA synthesis.

In infections, RA signaling may also induce the production of proinflammatory cytokines by DCs, promoting the differentiation of effector T cells [12] and enhancing the cellular activation state, in addition to the promotion of the formation of tertiary lymphoid structures [10]. These structures are formed in response to nonresolving inflammation generating lymphoid aggregates that drive adaptive immune reactions [54, 55]. RA also influences the maturation of monocyte-derived DCs (MoDCs) by increasing the expression of major histocompatibility complex (MHC) class II and CD86 and regulating the survival of DCs via the RAR*α*/RXR pathway [56]. In parallel to the activation of the innate immune response, RA promotes human DCs to induce IL-10-producing T cells to control inflammation and the maintenance of tissue homeostasis [57].

There are other sources of RA, such as lamina propria stromal cells, intestinal epithelial cells, and macrophages. Intestinal macrophages express RALDH1 and RALDH2, but that expression is dependent on external stimuli, such as cytokines and TLR ligands, whereas in CD103^+^ DCs, the expression of these enzymes appears to be related to dietary vitamin A [51, 58]. In contrast, atRA treatment upregulates CD103 expression in human MoDCs, increasing the ability of the DCs to synthesize RA [59] and inducing tolerogenic DCs.

Mucosal macrophages, which constitute the most abundant population of phagocytic cells in the gut, show inflammatory anergy, avoiding inflammation in normal intestinal mucosa despite proximity to immunostimulatory microbiota [58]. RA also acts directly on macrophages at both mucosal sites and other immunological sites. atRA modulates peritoneal macrophage activation by endotoxin and IFN-*γ* by suppressing TNF production and nitric oxide (NO) synthesis [60]. In addition, atRA inhibits the expression of PGE2 and COX-2 and the release of TNF, which are induced by bacterial lipopolysaccharide (LPS) in murine peritoneal macrophages [61].

Retinoid treatment inhibits IL-12 production in LPS-activated macrophages by inhibiting nuclear factor kappa B (NF*κ*B)-DNA interactions and the competitive recruitment of transcription integrators between NF*κ*B and RXR [62]. atRA also inhibits the LPS-induced production of the proinflammatory cytokines TNF-*α* and IL-12 and potentiates IL-10 production in the THP-1 monocyte/macrophage cell line and human cord blood mononuclear cells (CBMCs) [63]. Plasma factors, such as transforming growth factor- (TGF-) *β* and PGE2, in combination with RA, act synergistically with IL-4 synthesized by basophils to increase the sensitivity of macrophages to IL-4, which contributes to M2 macrophage polarization and the regulation of the inflammatory process in mice [64].

Tissue-resident macrophages are highly heterogeneous in terms of their functions and phenotypes as a consequence of adaptation to different microenvironments [65]. Monocyte-derived inflammatory macrophages can be converted into the resident tissue phenotype in a vitamin A-dependent manner [66]. VAD mice fail to convert tissue-resident macrophages during infection, which may lead to a deregulated inflammatory process [66].

In general, the impact of RA on macrophages suggests that RA inhibits the production of inflammatory cytokines and favors the generation of tolerance.

### 3.2. Modulation of Innate Lymphoid Cells (ILCs) by RA

ILCs constitute a group of tissue-resident innate immune cells that can regulate inflammation and repair tissues in multiple anatomical compartments, particularly on the barrier surfaces of the skin, airways, and intestine [67]. ILCs are derived from the same DNA-binding 2- (Id2-) dependent precursor and are characterized by the expression of the IL-7 receptor [68].

These cells are subdivided into the following three main groups: group 1 ILCs (ILC1), which include natural killer (NK) cells, are induced by transcription factor T-box expressed in T cells (T-bet) and produce IFN-*γ*; ILC2 require the GATA-binding protein 3 (GATA3) transcription factor and produce IL-5 and IL-13; and ILC3 depend on the transcription factor retinoic acid receptor-related orphan nuclear receptor gamma (ROR*γ*t) and secrete IL-17 and IL-22 [69].

ILC1 are accumulated during chronic inflammation in the gut (inflammatory bowel disease) and lung (chronic obstructive pulmonary disease), where they contribute to IFN-*γ*-mediated inflammation; ILC2 are mainly found in the lung but can also be present in the skin and gut, are related to helminth defense, and are mostly involved in tissue repair, allergy, and asthma, and ILC3 are implicated in gut barrier defense and skin inflammation [70].

In addition, ILC3 include lymphoid tissue-inducing cells (LTi) that contribute to the formation of secondary lymphoid organs [71]. RA during gestation is necessary for the development of fetal LTi cells during the embryonic stage, since maternal RA upregulates the ROR*γ*t transcription factor and favors the formation of lymphoid tissue, promoting greater efficiency in the immune responses of adult offspring [72]. Similarly, RA is required during the postnatal phase for the generation of intestinal ILC3 and LTi cells in adult mice, since its deficiency or the blockade of RA-RAR signaling reduces the development of enteric lymphoid tissue [73].

RA also induces the expression of *α*4*β*7 and CCR9 in ILCs 1 and 3, which is crucial during antiviral and antibacterial responses in the intestinal mucosa; this effect is not observed in ILC2 since the homing receptors expression of these cells is determined during development in the bone marrow [8]. RA associated with IL-2 *in vitro* contributes to the synthesis of IL-5 and IL-13 by ILC2 and IFN-*γ* by ILCs 1 and 3, which are important for the functional maintenance of ILCs in allergic and inflammatory diseases [74].

Intestinal tolerance induced by RA could be obtained by modulating ILC3 function in the GALT by increasing IL-22 production during colon inflammation induced by dextran sulfate sodium (DSS) or pathogenic bacteria, in mice [75]. In addition, the RAR receptor, which acts as a transcription factor, is able to bind the IL-22 promoter and directly promote its transcription. Moreover, human intestinal ILC1 can differentiate into ILC3 *in vitro* in response to IL-2, IL-23, IL-1*β*, and RA [59], which is important for tolerance.

In NK cells, RA acts tolerogenically and suppresses the human NK cell cytotoxicity activated by IFN-*α* [76]. NK cells are cytotoxic cells that act against tumor cells and virus-infected cells by a complex process of signaling mediated by activating and inhibitory receptors [77]. Additionally, antibody-dependent cellular cytotoxicity (ADCC) directs the cytotoxicity of NK cells toward antibody-coated target cells [78]. RA can influence the activity of NK cells by inhibiting ADCC and its natural cytotoxicity *in vitro* [79]. In addition, high concentrations of atRA inhibit NF*κ*B signaling in NK cells, negatively regulating the secretion of IFN-*γ*, which is important for granzyme B release [80]. *In vitro* treatment with *13-cis* RA also regulates NK cell activity by increasing CD158b, which is a killer inhibitory receptor [81].

On the other hand, RA increases the expression of MHC class I chain-related proteins A and B (MICA and MICB) in tumor cells that bind the natural killer group 2D (NKG2D) receptor in NK cells, promoting their activation [82]. In addition, the number of circulating NK cells in humans is positively regulated by the level of retinol stocks [83]. Thus, RA exerts a bidirectional effect on NK cells, which may contribute to its inhibition or activation.

### 3.3. Effect of RA on B Cell Differentiation

RA plays an important role in the humoral response and is essential for B cell production of IgA antibodies playing a multifactorial role in mucosal immunity [10, 84]. Oral administration of RA in VAD mice proved to be efficient in reestablishing IgA production after influenza vaccination [7]. In addition, vitamin A and zinc deficiency leads to a decrease of serum IgA and a drastic reduction of humoral mucosal immunity [85]. During vaccination, the association with RA potentiates the immune response in both adult and neonatal mice, suggesting an important role of RA as a vaccine adjuvant, especially during the early stages of life [86, 87].

Retinoids are described as important cofactors for the stimulation and proliferation of B cells, accelerating B cell lymphopoiesis [88, 89]. RA increases the number of peripheral B cells in the spleen while decreasing lymphoid progenitors in the marrow; these effects are mediated by an increase of the early B cell factor 1 (EBF1) and paired box protein-5 (Pax-5) transcription factors, which are crucial for B cell development [89]. Moreover, RA accelerates the maturation of human B cells and their differentiation into plasma cells [90].

The development of an effective long-lasting humoral response requires the formation of germinal centers (GCs) in the lymphoid follicles, where interactions between B cells and follicular helper T cells guarantee the development of memory B cells and long-lived plasma cells [91]. Thus, B cells undergo somatic hypermutation and immunoglobulin class-switching recombination as a part of the GC reactions mediated by the activation-induced cytidine deaminase (AID) enzyme [92]. RA may increase more differentiated B cell phenotypes by upregulating the expression of AID and B lymphocyte-induced maturation protein-1 (Blimp-1) and increasing the expression of CD138 and IgG in splenic B cells [93]. RA also induces the expression of interferon regulatory factor 4 (IRF4), which is involved in the generation of plasma cells and RA-mediated IgG production, favoring AID expression [94]. In addition, RA may increase IgM and IgG syntheses in human B cells from CBMCs and adult peripheral blood mononuclear cells (PBMCs), respectively [95].

The microenvironment may directly affect the modulation of the humoral response. The combined effects of bacterial products and RA on the intestinal mucosa trigger signaling cascades via TLRs and RAR, respectively, activating follicular dendritic cells (FDCs) [96]. This process enhances the synthesis of CXC chemokine ligand 13 (CXCL13), which is a chemoattractant of B cell follicles in lymphoid tissues, and increases the expression of B cell-activating factor (BAFF), which is an important factor for B cell survival and TGF-*β* [96]. Collectively, RA favors the migration and survival of B cells and leads to the preferential generation of IgA in the intestine [84]. Other components present in the mucosa contribute to the generation of IgA, such as lactoferrin [97], which, together with RA, leads to the production of IgA by peritoneal B-1 cells [98].

On barrier surfaces, the humoral response is the main effector response to frequent microbial challenges from both the host microbiota and the external environment. RA plays a key role in the modulation of mucosal inflammatory responses by contributing to the synthesis of antibodies, especially IgA, ensuring immunity and tolerance.

### 3.4. Effects of RA on the T Cell Population

The effects of RA on the balance of Th1/Th2 responses are controversial. Some studies indicate that high levels of RA can promote the differentiation of naïve T cells into Th2 cells by inducing IL-4 gene expression [1]. In addition, RA modulates IL-12 production by APCs, inhibiting Th1 cell differentiation [99], and induces the expression of GATA3 and signal transducer and activator of transcription 6 (STAT6), which is important for the maintenance of the Th2 response [1, 36]. RXR agonists and *9-cis* RA also favor the development of Th2 cells [100]. However, an experimental ovalbumin-induced asthma murine model suggests that vitamin A deficiency is related to increased pulmonary inflammation by inducing type 2 cytokines [101]. In addition, some studies have shown that oral vitamin A treatment (vitamin A supplementation diet) indirectly reduces pulmonary inflammation as a result of the anti-inflammatory effects of RA on other immune cells and Treg cell generation in the lung without directly affecting the Th2 population [18, 19].

Although RA inhibits Th1 responses, it is essential for the stability and maintenance of Th1 cells, repressing transcription factor ROR*γ*t, which is important for the induction of Th17 cells [102]. Furthermore, RA plays an important role in the maintenance of Th1 responses since VAD mice exhibit a negative Th1 response after infection with *Toxoplasma gondii* [12].

The impact of RA on the Th17/Treg balance has a known mechanism. Small intestinal lamina propria DCs synthesize RA and have the ability to generate Tregs in the presence of TGF-*β* [6]. Thus, elevated levels of TGF-*β* promote the generation of Tregs from naïve CD4 T cells by an RA-dependent mechanism [9, 39, 103, 104] in which atRA promotes the acetylation of histones on the promoter of the Foxp3 gene. In addition, atRA, which activates STAT6 through IL-4 signaling, also promotes the acetylation of histones on the Foxp3 gene promoter, increasing its expression in the cell [105, 106].

At the steady state, RA inhibits the differentiation of naïve T cells into Th17 cells by blocking IL-23 and IL-6 signaling [107]. RA also indirectly induces Treg conversion by inhibiting the CD44^hi^CD4^+^ T cell population of memory cells, which blocks the differentiation of naïve T cells into Tregs via the secretion of IL-4, IL-21, and IFN-*γ* [108]. In addition, RA controls the generation of T cells with an inflammatory profile in the GALT, suppressing the differentiation of naïve T cells into Th17 cells in the mucosa to maintain tolerance [45]. In contrast, IL-6 inhibits the generation of Tregs, favoring the expansion of Th17 cells in colitis [105, 109].

Th17 cells are generated in the presence of IL-6 and IL-21 and low levels of TGF-*β* in the intestinal mucosa, mainly during chronic inflammation [109, 110]. These cells can secrete cytokines, such as IL-17A, IL-17F, IL-21, and IL-22, which can control bacterial and fungal infections at mucosal sites [10]. Although RA inhibits Th17 generation, during infection of the intestinal mucosa, low concentrations of RA produced by TLR5^+^ lamina propria DCs induce the generation of Th17 cells, potentiating the protective response in the mucosa [111]. In addition, RA is essential for the in situ generation of Th17 cells in the intestinal mucosa during infection caused by *Toxoplasma gondii* [12].

Oral supplementation with RA in mice with chronic inflammation in the ileum may attenuate inflammation by restoring the balance between the Th17 and Treg populations, increasing the number of CD103^+^ DCs and RALDH2 expression by a positive feedback mechanism [45, 109]. Microbial stimuli, such as the TLR-2 receptor ligand in mice, also increase RALDH2 expression and RA production, promoting regulatory T cells and inhibiting the generation of Th17 cells [42, 52]. Thus, RA balances the generation of subsets of T cells depending on the conditions and factors of the microenvironment to maintain homeostasis.

## 4. Immunomodulatory Effect of RA during Inflammatory Processes

Inflammation during immune responses is an important way to remove tissue injuries and promote restoration. Inflammation can occur as a physiological process in which dead cells are removed from tissues, keeping the tissues healthy, but it can also be caused by several other stimuli, such as pathogen infections, damaged cells, toxic compounds, or irradiation [112].

Typically, organisms undergo acute inflammatory responses in which molecular and cellular interactions resolve injury and infections without tissue damage, contributing to the restoration of tissue homeostasis. However, uncontrolled acute inflammation or nonresolution of infection may generate chronic inflammation, contributing to a variety of chronic inflammatory diseases [113, 114].

**Figure 3:**
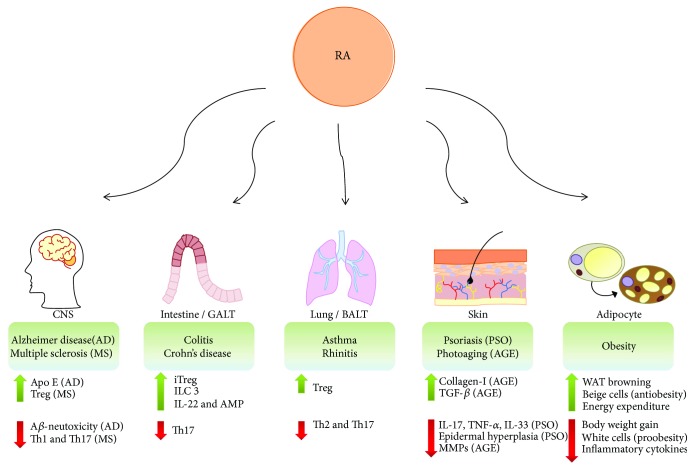
Potential anti-inflammatory effects of RA. RA can decrease inflammatory processes, promoting homeostasis and attenuating harmful inflammatory responses in mucosa and tissues. RA shows immunosuppressive effect on Th1/Th17 cells in multiple sclerosis (MS) and induces Apo E in microglia, protecting from the neurotoxic effects mediated by amyloid *β* (A*β*) in Alzheimer disease (AD), contributing to neuronal homeostasis. RA is crucial for intestinal tolerance, by inducing Treg, cytokines IL-10 and IL-22, and antimicrobial peptide (AMP) synthesis, which may lead to Th17 inhibition. RA modulates inflammatory airway diseases (asthma and rhinitis) by inhibiting Th2/Th17 response and enhancing Treg cells. Retinoids increases type I collagen and TGF-*β*, reducing matrix metalloproteinase (MMPs) in photoaging (AGE), and reduces IL-1 family cytokines (IL-17 and TNF-*α*), IL-33, and epidermal hyperplasia in psoriatic (PSO) lesions. RA also has effects in adipocytes, promoting white adipose tissue (WAT) browning by differentiation into beige cells (antiobesity) instead of white cells (proobesity). The formation of brown adipocytes within WAT enhances energy expenditure and reduces obesity. In addition, RA can repress the expression of inflammatory chemokines and cytokines, inhibiting inflammatory responses triggered by obesity.

Simultaneously, some components of the diet act as anti-inflammatory mediators by attenuating acute and chronic inflammatory processes, promoting homeostasis and, thus, ameliorating the harmful effects of inflammatory responses. Here, we focus on how RA modulates the inflammatory response at different mucosal sites and different tissues (Figure 3).

### 4.1. Intestinal Mucosa

Inflammatory bowel disease (IBD), which is mainly referred to as Crohn's disease and ulcerative colitis, is a result of chronic inflammation characterized by excessive innate immune cells activation, tissue damage, and the induction of adaptive immune responses against the intestinal microbiota. Gut dysbiosis, mainly among commensal bacteria, initiates an exacerbated inflammatory response. This disorder can be caused by multiple factors, including abnormal immune responses, genetic susceptibility, infection, dietary habits, and the administration of antibiotics [115, 116].

RA has been shown to regulate immune responses and restore the Th17/Treg balance, mainly in the intestinal mucosa, showing that RA plays an important role in intestinal mucosal homeostasis [45, 109]. Vitamin A impairs the reprogramming of inducible Tregs (iTregs) into Th17 cells during intestinal inflammation induced in a T cell-dependent colitis model [13, 14]. In addition, DCs were more efficient for Treg differentiation after the restoration of intestinal RA by diet in intestinal tumor models [14].

In an experimental model of DSS- or pathogenic bacteria-induced colitis, RA was shown to attenuate inflammation by increasing IL-22 production by ILC3 and T*γδ* cells and, consequently, increasing the synthesis of antimicrobial peptides [75] or by decreasing TNF levels and NF*κ*B activation [115]. Moreover, in inflamed intestinal tissues from Crohn's disease patients, the number of CD127^+^ ILC1 increased at the cost of ILC3 [59]; however, ILC1 can be differentiated into ILC3 *in vitro* and *in vivo* upon IL-2, IL-23, and IL-1*β* stimulation and this process was enhanced in the presence of RA, reducing inflammation [117]. In addition, *in vitro* atRA treatment of inflamed colonic mucosa from patients with ulcerative colitis and colitis-associated cancer modulates the LPS/TLR4/NF*κ*B signaling pathway and decreases nitric oxide synthase 2 (NOS2) and TNF-*α* expression [118]. This finding suggests that RA may be a target for future colorectal cancer treatments.

The absence of RA in VAD mice makes them more susceptible to the development of DSS-induced colitis and colon cancer due to worsening of chronic inflammation in the intestine [119]. In this context, CYP26B1, which is responsible for the catabolism of RA [35], has been shown to regulate the differentiation and function of CD4 T cells during experimental colitis, driving the cells towards an inflammatory profile. The passive transfer of naïve cytochrome p450 family 26 subfamily b1-deficient (Cyp26b1^−/−^) mice CD4 T cells into recombination-activating gene 1-deficient (Rag1^−/−^) mice resulted in a significantly reduced disease state in a model of T cell-dependent colitis [120].

In humans and murine models of ulcerative colitis associated with colorectal cancer, alterations of atRA metabolism mediated by microbiota-induced intestinal inflammation, with increasing levels of CYP26A1, another atRA-catabolizing enzyme, reduced colonic atRA and promoted tumorigenesis [121]. Supplementation with atRA reduced the tumor burden, and this effect was mediated by cytotoxic CD8 T cells activated by MHC I upregulation on tumor cells [121]. In a case-control study with 898 colon cancer cases, 501 rectal cancer cases, and 1399 matched controls, an association between higher plasma retinol concentrations and a lower risk of colon cancer was observed, mainly in proximal colon cancer [122]. This evidence suggests a role of RA in the prevention of colon cancer.

Serum retinol levels in adults and children with Crohn's disease are lower than those in healthy people probably due to a deficiency in nutrient absorption [123, 124]; similar results were reported for ulcerative colitis [125]. Considering the tolerogenic role of RA in the intestinal mucosa and the fact that IBD patients have low levels of serum retinoids, RA administration should be an adjuvant treatment for inflammatory diseases.

Moreover, we must consider that RA also plays an important role during intestinal inflammation caused by pathogen infection, as observed during *Salmonella typhimurium*-mediated gastroenteritis in mice [126] and during *Vibrio cholera* infection after pretreatment with RA prior to immunization [127].

Overall, RA displays an important anti-inflammatory activity in the control of inflammation in the intestinal mucosa, but more studies are necessary to better understand the role of RA in inflammatory processes.

### 4.2. Airways and the Lung

Inflammatory airway diseases, such as asthma and allergic rhinitis, have a high prevalence around the world. Airway inflammation is mediated by Th2 cells that characteristically produce IL-4, IL-5, and IL-13 [128]. Moreover, in chronic airway inflammatory diseases, the massive infiltration of eosinophils is mediated by allergen-specific Th2 cells and neutrophils also participate in chronic obstructive pulmonary disease mediated by Th17 cells [129].

The role of RA in the pulmonary mucosa is controversial. Supplementation with vitamin A has been shown to increase the severity of asthma in experimental models with high levels of IgE and IgG1 antibodies and pulmonary inflammation [29]. In addition, RA has the ability to induce Th2 responses and inhibit Th1 responses [99, 100]. In contrast, oral administration of Net-4IB, an RXR partial agonist, suppressed aryl hydrocarbon receptor (AHR) and inflammatory cell accumulation in the airways and attenuated TNF-*α* levels in the lung and IL-5, IL-13, and NO levels in bronchoalveolar lavage fluid from mice [130]. The RXR partial agonist Net-4IB may be a promising candidate for the treatment of allergic airway inflammation.

Moreover, vitamin A deficiency has been related to an increased asthma incidence in children due to damage to the pulmonary mucosa and to the maintenance of the airway epithelium [131]. During vitamin A deficiency, a mouse asthma model revealed the induction of Th2 cytokines, such as IL-5 and IL-13, and an increase in pulmonary inflammation [101]. The administration of RA may attenuate inflammation by increasing the population of regulatory T cells in the lung and decreasing the tissue damage caused by inflammation [18, 19]. The association between RA and ovalbumin in oral tolerance in a murine model of bronchial asthma efficiently inhibited the inflammatory response and decreased eosinophilic infiltration besides Treg cells induction in the lung [132]. Treatment with atRA was able to attenuate airway inflammation by inhibiting Th2 and Th17 cytokines and downregulating the expression of the GATA3 and ROR*γ*t transcription factors in the lung [128]. Similar results were observed in a murine model of allergic rhinitis [133]. Interestingly, lung-resident tissue macrophages that coexpress TGF-*β* and retinal dehydrogenases (RALDH1 and RALDH2) are able to induce Treg cells at a steady state, favoring airway tolerance [134].

In human studies, the retinoid concentrations in the serum were significantly lower in patients with asthma than those in healthy control subjects and were even lower in patients with severe asthma than those in patients with mild asthma [135, 136], highlighting the importance of equilibrating physiological RA concentrations in airway diseases.

### 4.3. Central Nervous System

Although the brain is an immunologically privileged site, in pathologic conditions of the central nervous system (CNS), an organized immunologic response can develop within the CNS to eliminate inflammation without tissue damage [137]. However, in some cases, a persistent inflammatory response develops during neurodegenerative processes, such as multiple sclerosis (MS) and Alzheimer's disease (AD).

MS is an autoimmune disease characterized by recurrent episodes of demyelination and axonal lesions mediated by Th1 and Th17 cells, macrophages, and immune inflammatory mediators [138]. Taking into account the immunosuppressive role of RA for Th1/Th17 cells and macrophages, it is not unreasonable to think that RA may exert beneficial effects in MS.

Indeed, treatment with atRA alone or in combination with calcitriol (an active vitamin D metabolite) in murine autoimmune encephalomyelitis (EAE), which is an experimental model of MS, increased the expression of the Foxp3 and TGF-*β* genes in splenocytes while reducing ROR*γ*t gene expression [139]. PBMCs from patients with MS who were supplemented with vitamin A for 6 months also showed an upregulation of TGF-*β* and Foxp3 gene expression [15] and a reduction in IFN-*γ* and T-bet gene expression [16]. RA treatment also suppresses T*γδ* cell pathogenic activity by decreasing IL-17 production, which is important for the maintenance of EAE [17]. Moreover, the combination of atRA and atorvastatin, which is a lipid-lowering agent with anti-inflammatory, immunomodulatory, and neuroprotective properties, causes the regression of the clinical and neuropathological features of EAE with reduced secretion of IL-17 and increased production of IL-10 and Foxp3^+^ Treg cells in splenocytes [140].

In MS, activated astrocytes participate in promoting lesion progression by secreting proinflammatory mediators and chemokines. In cocultures with inflamed endothelial cells, primary astrocyte-derived RA attenuated oxidative stress [141]. In addition, murine astrocytes that were stimulated with LPS and treated with atRA expressed no or very low levels of CCL and CXCL chemokines [142].

All these data suggest that retinoids are candidates for the treatment of neuroinflammation. Indeed, the use of RA-loaded polymeric nanoparticles (RA-NPs) modulates the murine microglial response towards an anti-inflammatory and neuroprotective phenotype (M2-like) in organotypic hippocampal slice cultures [143]. Recently, the intravenous administration of RA-NPs was shown to prevent ischemic injury in the immature brains of 2-day-old mice, demonstrating the role of RA in the control of neuroinflammation [144].

RA also has neuroprotective activity and is capable of increasing barrier tightness in human-induced pluripotent stem cell-derived brain endothelial cells by RAR*α*, RAR*γ*, and RXR*α* activation [145]. In addition, treatment with RA in experimental models of AD and *in vitro* was beneficial.

AD is a progressive neurodegenerative disease characterized by neuroinflammation with reactive microglia, astrogliosis, proinflammatory cytokines, amyloid-*β* (A*β*) peptide deposition, and progressive memory loss [146]. Treatment with RA in an experimental model of AD was beneficial by inhibiting microglial activation in the hippocampus and improving the proliferation of stem cells [147], as well as increasing the synthesis of apolipoprotein E (Apo E) in human macrophages [148]. Apo E acts on microglia, protecting them from the neurotoxic effects of amyloid *β*, and contributes to neuronal homeostasis [149].

Oral coadministration of Am80 (an RAR-*α*/*β* agonist) and HX630 (an RXR agonist) reduced the level of insoluble A*β* peptide in the brain by promoting the differentiation of IL-4-responsive M2-like microglia and increasing their activity for the clearance of oligomeric A*β* peptides in an experimental model of AD. This finding showed that combination treatment with RAR and RXR agonists could be an effective approach for AD therapy [146].

atRA administration prevents LPS-induced neuroinflammation, NO production, amyloidogenesis, and memory impairment in aged rats [150]. PBMCs from patients with AD in cultures with atRA showed downregulated spontaneous NO production and iNOS expression, which was associated with a reduction in IL-17A production and increased IL-10 release [151].

All these data suggest that RA may be a potential target in both MS and AD treatments.

### 4.4. The Skin

The skin is the primary barrier that provides protection against microbial pathogens and physical and chemical insults to organisms [152]. The skin is composed of the following layers: epidermis, basement membrane, dermis, and subcutaneous fatty region. Each layer has several structures, such as hair follicles, sweat glands (in humans but not mice), sebaceous glands, nerves, blood vessels, and lymphatics. The epidermis and dermis have a variety of cell types, including immune cells. Together, these cells form an orchestrated defense against invading pathogens [153]. In the epidermis, in addition to melanocytes that produce melanin and keratinocytes, there are Langerhans cells, which are the main skin-resident immune cells, and are more involved in tolerogenic than inflammatory responses [154]. The other types of immune cells, such as DC subpopulations, macrophages, ILC2, NK, and B and T cells, reside in the dermis [155].

In the epidermis, keratinocytes also play an important role in defense against pathogens. Epidermal keratinocytes are proinflammatory effector cells with a large production of antimicrobial peptides (AMPs), proinflammatory cytokines, and chemokines [155]. Keratinocytes also express TLRs [156], which are crucial for promoting skin immune responses and Th1 responses [152]. However, an imbalance in the immune response and microbiota or persistent infections can generate skin inflammations, causing several diseases.

The use of retinoids has long been established for the treatment of immune-mediated skin diseases. In dermatological treatment, retinoids are typically classified into three generations according to how they were developed [157]. The first-generation retinoids are the naturally occurring nonaromatic retinoids, including retinol, retinal, isotretinoin (*13-cis* RA), tretinoin (atRA), and alitretinoin (*9-cis* RA). The second-generation retinoids are the monoaromatics (etretinate, acitretin, and motretinate). The third-generation retinoids are the polyaromatics (bexarotene, adapalene, and tazarotene) [157, 158].

Photoaging is a process mainly triggered by ultraviolet radiation from chronic sun exposure that leads to DNA damage and the production of reactive oxygen species, which both promote inflammation [159] and result in increased matrix metalloproteinases (MMPs) and collagen degradation [160]. Retinoids have demonstrated efficacy in the treatment of the photoaged skin. The effects of RA include the inhibition of the expression of MMPs [161]; inhibition of tyrosinase activity, which increases epidermal cell turnover and leads to increased shedding of melanin-laden keratinocytes; reduction of inflammatory cytokine production; and enhancement of type 1 collagen and TGF-*β* [162]. All these effects contribute to the improvement of symptoms in photoaging.

In addition to aging/photoaging, the application of retinoids in skin diseases is very diverse and retinoids have been used in treatments for acne, rosacea, psoriasis, lichen planus, basal cell carcinoma, and so on [158]. Acne vulgaris is a common chronic inflammatory cutaneous disease that involves the pilosebaceous unit with abnormal keratinization leading to follicular plugging [163]. Retinoids act by increasing the turnover of follicular epithelial cells and accelerating the shedding of corneocytes, which helps normalize keratinization. Retinoids also exert a sebum-suppressive effect following oral isotretinoin administration [164]. The use of isotretinoin also induces the remission of acne by normalizing the innate immune response to the commensal bacterium *P. acnes* [165]. This remission occurs due to decreased monocyte TLR-2 expression and the subsequent inflammatory cytokines response to *P. acnes.* Combining retinoids with other components and antibacterial agents can decrease irritation and increase the efficacy of retinoid treatment [163, 166]. Importantly, retinoids regulate the transcription factor AP-1, resulting in the inhibition of MMPs, which are responsible for scar formation in acne [165].

Psoriasis is a prototype inflammatory skin disease characterized by marked keratinocyte hyperproliferation and altered differentiation associated with dermal and epidermal infiltration of leukocytes [166]. Tazarotene is the most commonly used retinoid for topical treatment [167–169] and is usually used in combination with phototherapy, corticosteroids, vitamin D, and other treatments [170–172]. Tazarotene acts by reducing plaque elevation and inflammation probably due to its anti-inflammatory role in immune cells [158].

In general, the use of retinoids in immune-mediated skin diseases has been highly beneficial for the patients.

### 4.5. Obesity

Obesity is a global health issue, and overnutrition and excess bodyweight are associated with an increased risk of developing metabolic disorders, such as diabetes and cardiovascular diseases. Several inflammatory markers have been consistently associated with obesity, suggesting that persistent low-grade inflammation is present in obesity [173, 174].

RA also plays an important role in the modulation of inflammatory processes at other sites and in other tissues. In human adipocytes, atRA represses chemokine and inflammatory cytokines expression by inhibiting NF*κ*B signaling. Since inflammatory responses triggered by obesity play a major role in the onset of insulin resistance, atRA supplementation may represent a preventive nutritional strategy for controlling obesity and its complications [175].

Besides the liver, adipose tissue contains a substantial amount of retinol and its metabolites [176]. The RARs are highly expressed in adipose tissues; therefore, the RARs are directly influenced by atRA [177]. atRA and *9-cis* RA, *in vitro*, inhibit proliferation and induce apoptosis in a human preadipocyte cell lineage [178]. In addition, RA enhances lipid oxidation and inhibits lipid's biosynthesis capacity [178], as well as, decreases body weight gain in an obese rat model independent of Stearoyl-CoA desaturase 1 (SCD1) gene regulation, which is an enzyme involved in the biosynthesis of monounsaturated fatty acids [179].

RA also promotes the remodeling of white adipose tissue (WAT) [180], which is associated with metabolic disorders. In a VAD model, a marked increase in adiposity and hypertrophy of WAT was observed [181]. In addition, RA induces white adipose tissue browning by increasing adipose vascularity, which promotes the differentiation into beige cells (antiobesity) instead of white cells (proobesity) [182]. The formation of brown adipocytes within WAT enhances energy expenditure, reduces obesity, and could help improve metabolic health [183].

RA treatment of obese mice induces RAR target genes involved in the regulation of lipid homeostasis, leading to the suppression of obesity and insulin resistance [184].

Moreover, in an experimental obesity model, RA produced by DCs and macrophages upon IL-13 stimulation from IL-33-activated islet-resident ILC2 cells induced insulin secretion by *β* cells [185]. The IL-33-ILC2 axis was activated after acute *β* cell stress but was defective during chronic obesity. However, the fact that RA increases insulin secretion shows its potential modulatory role in metabolic diseases.

## 5. Conclusion and Future Perspectives

Globally, more than 2 billion people are affected by micronutrient deficiency and at least half of children aged 6 months to 5 years worldwide suffer from one or more micronutrient deficiencies. Vitamin A deficiency is a public health concern, and vitamin A supplementation in children is highly effective in reducing mortality from all causes. In adults, the anti-inflammatory effects of RA, which favor immune homeostasis, are a treatment strategy alone or in association with other drugs for inflammatory intestinal diseases, neurodegenerative processes, skin aging, and cancer. Furthermore, alterations of serum RA levels are not only indicators of homeostasis disequilibrium but also biomarkers for the intestinal inflammatory process. An uncontrolled vitamin supply and micronutrient deficiencies reinforce the need to better understand the effects of RA on the immune system and inflammatory diseases.
